# Greater occipital nerve stimulation for cognitive impairment in Parkinson's disease: A two-stage feasibility and tolerability study

**DOI:** 10.12688/hrbopenres.14230.1

**Published:** 2025-11-21

**Authors:** Liam Kennedy, Ananya Sanagavaram, Elva Arulchelvan, Sven Vanneste, Iracema Leroi

**Affiliations:** 1 Trinity College Dublin School of Medicine, Dublin, Leinster, D02 PN40, Ireland; 2 Tallaght University Hospital, Dublin, Leinster, Ireland; 3 Trinity College Dublin School of Psychology, Dublin, Leinster, Ireland; 4 Global Brain Health Institute, County Dublin, County Dublin, Ireland

**Keywords:** Neuromodulation, Cognitive impairment, Parkinson’s disease, Feasibility

## Abstract

**Background:**

Effective treatments for cognitive impairment in Parkinsons’s disease (PD) are needed, with Greater Occipital Nerve Stimulation (GONS) emerging as a potential therapeutic approach. This study aimed to explore PD patients' attitudes toward GONS and assess its safety and tolerability in an open-label field study.

**Methods:**

A two-stage study was carried out, comprising an online survey to gather self-reported attitudes from individuals with PD toward GONS as a treatment option, followed by an open-label field study evaluating the safety, tolerability, and acceptability of GONS in PD-MCI patients.

**Results:**

Of 50 survey participants, 86% expressed willingness to try GONS for cognitive impairment. Five participants completed the GONS intervention, which was found to be safe, well-tolerated, and acceptable

**Conclusion:**

People with PD are open to novel interventions for cognitive impairment. GONS was shown to be safe and well-tolerated, warranting further investigation as a potential therapeutic approach.

## Introduction

Parkinson’s disease (PD) is a progressive neurodegenerative condition and a significant source of global neurological disease burden, with incidence rates increasing over the past 20 years ^1^. While often characterised primarily as a motor syndrome, non-motor symptoms, including cognitive impairment, contribute significantly to the disability arising from PD, with between 60–80% of people with PD experiencing cognitive impairment that impacts their quality of life ^2^. Studies have shown that patients may find non-motor symptoms, including cognitive impairment, more disabling and more difficult to address with medication than traditional motor symptoms ^3^.

Mild cognitive impairment (MCI) may be a presenting feature of PD or may develop over the course of the disease. Studies have found that between 25–45% of non-demented people with PD meet Movement Disorder Society criteria for PD-MCI ^4, 5^. Of PD patients who survive 10 years, 46% will develop PD dementia (PDD), a prevalence that rises to 83% in those who survive 20 years ^6^. Thus, cognitive impairment in PD represents an important target of research in terms of disease burden, and PD-MCI may represent a pivotal interventional stage for slowing or preventing the progression of impairment to the point of dementia.

Licensed treatments for cognitive impairment in PD are limited. Rivastigmine, an acetylcholinesterase inhibitor, is licensed for symptomatic treatment in PDD but lacks evidence of benefit in the PD-MCI group ^7^. Trials of other pharmacological agents, including rasagiline and atomoxetine, have failed to provide evidence of objective benefit in PD-MCI ^8, 9^. A recent Cochrane review concluded that there is an urgent need for more research in non-pharmacological interventions to address cognitive impairment in PD ^2^.

The locus coeruleus (LC) is a brainstem nucleus and the primary source of noradrenergic transmission throughout the brain ^10^. It has roles in arousal, cognition and learning ^11^, and the role of LC degeneration in PD dementia has been long established ^12^. PD patients with MCI have been demonstrated to have significantly more evidence of LC dysfunction than those without, as measured by neuromelanin-sensitive MRI ^13^. LC degeneration in PD patients correlates specifically with cognitive, as opposed to motor, symptoms ^14^. Neuromodulatory interventions, including transcranial direct current stimulation (tDCS), repetitive transcranial magnetic stimulation (rTMS), and invasive deep brain stimulation (DBS), have been explored in PD-MCI. Trials to date have been heterogeneous in terms of patient selection, stimulation protocol, outcome measurement, and results. Where results have been positive, these have been inconsistent across studies, with some finding modest benefit in single cognitive domains or tests only ^15, 16^. A 2022 review concluded that neuromodulation for cognitive impairment in PD held promise, but that further work in identifying the most effective and well-tolerated targets was needed ^17^.

As interest in non-pharmacological interventions increases, novel neuromodulatory approaches have been described. Targeting peripheral afferent nerves to modulate central nervous system neurotransmission and plasticity has emerged as a potentially promising approach. Transcutaneous vagus nerve stimulation (VNS) has demonstrated efficacy in enhancing associative memory in older adults ^18^. In a novel randomized control trial by Luckey et al., greater occipital nerve stimulation (GONS) utilizing tDCS has similarly been shown to enhance performance of older adults in an associative memory task, with benefits persisting for up to 28 days ^19^. GONS is hypothesized to enhance cognitive performance by causing downstream excitation of the LC, with an associated stimulation in noradrenergic transmission in the pons, hippocampus, and amygdala. Improvement in associative memory following GONS was associated with increased levels of salivary α-amylase levels, suggesting that increased noradrenergic activity via the locus coeruleus mediated the effect of GONS on associative memory ^19^. Given the demonstrated link between locus coeruleus dysfunction and decreased noradrenergic transmission in Lewy body-related cognitive impairment, this stimulation may offer greater therapeutic potential in PD-related cognitive impairment than in other neurodegenerative neurocognitive disorders such as Alzheimer’s dementia ^7, 20^.

To ascertain the acceptability and initial feasibility of GONS for PD-MCI, we undertook a two-part study. The first part involved a wide-ranging survey to explore knowledge, attitudes, and experiences of individuals with PD in relation to non-pharmacological interventions for cognitive impairment. The second part was an open-label pilot feasibility trial involving a purposive sub-sample of five survey respondents to assess the safety, tolerability, and acceptability of GONS as a potential therapeutic intervention for individuals with PD-MCI.

## Materials and methods

### Stage 1: Patient survey of acceptability

***Recruitment and participant inclusion***

Participants were self-selected individuals living with PD or Lewy body disease, recruited via online newsletters published by Parkinson’s Ireland and the "Get Involved with Research" page of Parkinson's UK. Eligibility criteria required participants to be over 18 years old, capable of providing digital consent, and willing to complete an email-based survey. Interested individuals emailed the research team, forming a mailing list through which the survey link, participant information leaflet, informational poster on GONS, and consent form were distributed. Reminder emails were sent two weeks after the initial contact to encourage responses. All participants completed written informed consent forms digitally.

***Survey design and data collection***

An anonymized, cross-sectional survey was employed, developed through consultations with the Dementia Research Group and informed by literature reviews. The final 32-item questionnaire included 26 multiple-choice, 4 open-ended, and 2 rank-order questions. These were grouped into six sections: demographics, health information, cognition-related views and knowledge, treatment perspectives, attitudes toward GONS, and an informed consent review. Data were collected using Microsoft Forms, ensuring compliance with institutional data protection policies.

***Outcome measures***

Key survey sections assessed the impact of cognitive issues in PD and explored attitudes toward cognitive symptoms, current management practices, and the use of non-drug therapies. Responses were measured using Likert-style scales for structured feedback. The survey also evaluated participant understanding of GONS, following their review of an informational video and poster. Open-ended questions encouraged additional insights, while rank-order questions prioritized concerns about risks and factors influencing non-drug treatment preferences.

***Data analysis***

Quantitative data were analysed using IBM SPSS Version 27, New York, USA. Qualitative responses underwent thematic analysis, including initial coding, formal coding, and thematic identification, conducted by two independent authors. The coding was conducted independently, followed by a cross-checking process to refine the coding framework and consensus was reached through discussion.

### Stage 2: Open-label field study

***Recruitment and eligibility***

Participants were recruited from the "Mind and Movement" clinic, a subspecialist centre for cognitive and neuropsychiatric disorders associated with Lewy Body diseases. Inclusion criteria targeted patients aged >50 years with a diagnosis of Lewy Body-related MCI. Exclusion criteria included comorbid neurocognitive disorders, recent seizures, current psychiatric disorders, prior neurological insults, substance misuse, or enrolment in other investigational studies. Eligibility was confirmed via telephone pre-screening. All participants completed written informed consent forms in person before commencing in the study.

***Intervention protocol***

The intervention followed an open-label, single-arm adaptation of a previously described sham-controlled study in older adults ^21^. Participants attended three visits:

•    Visit 1 (Day 1): Baseline assessments included demographic data collection and pre-intervention ratings of mood, anxiety, and cognition. A paired English-Swahili word association task was introduced, involving a study phase (memorization of paired words) and a test phase (recall of English translations).

•    Visit 2 (Day 7): Participants repeated the test phase of the word association task.

•    Visit 3 (Day 28): The test phase was repeated, alongside reassessments using cognitive rating scales.

    The GONS stimulation comprised application of non-invasive tDCS to the area of scalp overlying the course of the greater occipital nerve bilaterally. A constant 1.5mA current was transmitted through saline-soaked sponges for a period of 250 seconds. Stimulation was delivered via a small, portable current stimulator with a maximum output of 10mA.

***Outcome measures***

Primary measures included the safety, tolerability, and acceptability of the intervention, assessed through an adverse events questionnaire ^22^ and post-intervention semi-qualitative surveys. Secondary exploratory measures encompassed mood and anxiety ratings (Beck’s Depression and Anxiety Inventories), cognitive performance scores (Mini Mental State Examination, Controlled Oral Word Association Test, California Verbal Learning Test II, Denis Kaplan Color-Word Interference Test, and digit span test), and performance on the English-Swahili task. All proprietary research instruments were used under an institutional copyright license.

***Data collection and analysis***

Demographic data, rating scales, and adverse event reports were collected manually and digitally encoded. Performance data for the word association task were automatically captured. Post-intervention feedback was gathered via telephone surveys and included Likert-scale evaluations of safety, tolerability, and acceptability. Quantitative analyses were conducted using SPSS, while qualitative feedback was analysed thematically.

## Results

### Stage 1: Patient survey of acceptability

***Participant characteristics***

A total of 65 prospective participants expressed interest in the study, with 50 completing the survey ( Table 1). All respondents self-reported a diagnosis of PD or Lewy Body Disease (LBD) and were members of patient support groups, including Parkinson’s Association Ireland and Parkinson’s UK. No survey data were excluded from analysis.

**Table 1.** Clinical and demographic characteristics of survey respondents.

	Survey Respondents N (%)
Mean age ± SD	63.62 ± 8.30
Median age	64
Female	17 (34%)
**Living status**	
Living alone	8 (16%)
Living with family	42 (84%)
**Education**	
Second level	20 (40%)
Third level	11 (22%)
Post-graduate	19 (38%)
**Employment**	
Employed (full-time)	8 (16%)
Employed (part-time)	3 (6%)
Unemployed	1 (6%)
Medical benefits (full-time)	8 (16%)
Medical benefits (part-time)	1 (2%)
Retired	29 (58%)
**PD clinical features**	
Mean age at diagnosis ± SD	59.44 ± 9.18
Mean no. months with PD symptoms ± SD	63.12 ± 43.83
Modified Hoehn-Yahr stage 1 ^1^	31 (62%)
Modified Hoehn-Yahr stage 2 ^2^	12 (24%)
Modified Hoehn-Yahr stage 3 ^3^	6 (12%)
Modified Hoehn-Yahr stage 4 ^4^	1 (2%)
Modified Hoehn-Yahr stage 5 ^5^	0
**PD treatments**	
Prescribed 1 PD medication	16 (32%)
Prescribed 2 PD medications	25 (50%)
Prescribed 3 PD medications	5 (10%)
Prescribed 4 PD medications	4 (8%)
Deep brain stimulation	2 (4%)
Dopamine agonist patch	2 (4%)
Continuous intestinal pump	1 (2%)

^1^Symptoms on one side only; ^2^Symptoms on both sides with balance intact; ^3^Symptoms on both sides, mild balance problems, can mobilise unassisted; ^4^Symptoms on both sides, severe balance problems; ^5^Significant symptoms on both sides, wheelchair user or requires assistance to transfer from bed

The cohort was predominantly male (66%), with the majority possessing a baseline educational qualification of secondary education or higher. The mean age at diagnosis was 63.62 ± 8.30 years, and the average duration of PD across participants was 63.12 months (approximately 5.26 years). Most respondents reported using two medications for PD management, and 86% identified themselves as being in stage 1 or stage 2 of the modified Hoehn-Yahr scale.

Regarding other complex interventions, two participants reported undergoing advanced procedures: one had received deep brain stimulation, and another was using a rotigotine patch.

***Survey results***

Based on the survey results, 84% of participants regarded non-drug treatments as important for addressing cognitive and thinking issues in PD. Additionally, there was broad acceptance of the GONS device across the participant cohort. Acceptance was evaluated through four key domains: participant-reported acceptability (83.7% agreement), perceived safety of the treatment (68%), willingness to try GONS (86%), and advocacy for making the treatment accessible to potential beneficiaries once validated by further studies (94%) ( Table 2).

**Table 2.** Acceptability of GONS treatment.

	N (%)
GONS is an acceptable treatment for thinking and cognition in PD	41 (83.7%)
GONS is a safe treatment for thinking and cognition in PD	34 (68%)
I would consider GONS treatment for myself for thinking and cognition in PD	43 (86%)
If the GONS treatment is effective in improving thinking and cognition in PD I believe it should be made available to patients	47 (94%)
I feel I have a good level of knowledge of the potential risks and benefits of the GONS device	39 (78%)
I feel I have limited knowledge of the potential risks and benefits of the GONS device	11 (22%)

GONS = greater occipital nerve stimulation; PD = Parkinson’s Disease

Participants ranked the primary inconveniences associated with using a non-drug treatment like GONS. The most concerning issues were wearing a device with wires connecting to electrodes on the skin, followed by the need to put on and take off the device daily, and experiencing unnatural, though not painful, sensations from stimulation. In contrast, the least concerning inconveniences were returning to the clinic periodically for treatment sessions and follow-up evaluations and learning how to integrate the device into their existing strategies for managing cognitive issues.

Factors influencing participants’ willingness to consider GONS as a non-drug treatment highlighted the importance of safety, the side-effects profile, and the qualifications and experience of the treating clinician. Rapid onset of benefits and whether the treatment was administered at home or in a clinical setting were ranked lower in priority. The descriptive quantitative data on ranked preferences are further explored in the qualitative analysis section. Significant differences in participants’ evaluations of inconveniences and risks associated with GONS were assessed using Friedman ANOVA by ranks.

***Qualitative analysis***

**Theme 1: Cognitive impairment in PD as an unaddressed need**

Participants expressed frustration that cognitive symptoms were often overlooked or considered secondary to motor symptoms. One respondent remarked, “They think of PD as a movement disorder and not so much as a cognitive condition.” Many participants reported significant anxiety about future cognitive decline, with some expressing dissatisfaction with the limited treatment options currently offered. One participant stated, “I should be given alternatives to try out... any other treatments,” while another felt unsupported, commenting, “Neither my GP nor my neurologist contribute any advice to help me make decisions with my Parkinson’s.”

**Theme 2: Acceptability of the intervention**

There was widespread openness and interest in GONS as a potential treatment for cognitive symptoms. Participants’ enthusiasm ranged from curiosity, such as “I would certainly try it out,” to strongly positive reactions, including “I feel this would be really helpful to those who are working full-time and need to function effectively.” Perceived safety and ease of use were seen as advantages, with some participants expressing confidence in the intervention’s safety due to its ethical approval for study. Others cited previous positive experiences with non-invasive nerve stimulation, which contributed to their interest in GONS. However, some participants expressed reservations, recalling unpleasant experiences with nerve conduction tests or perceived negative effects of electroconvulsive therapy. Despite these concerns, a cautious optimism prevailed, typified by a participant who stated, “If this treatment is as gentle as it seems, maybe it could do some good.”

**Theme 3: Queries regarding the ONS treatment process**

Participants were keen to obtain more information about the GONS treatment process. Questions centred on the frequency, duration, and application site of treatment sessions, as well as the feasibility of wearing the device during daily activities, such as cooking or cleaning. Concerns about long-term efficacy were also prominent, with participants asking whether the effectiveness of GONS might decline over time. Many sought clarity on the functionality and maintenance of the device, eligibility criteria, and the speed of cognitive improvement. Some asked whether the treatment was primarily preventive or curative, as exemplified by the question, “Does it have a preventive impact or is that known yet?”

**Theme 4: Queries regarding safety and side-effects of ONS**

Safety concerns emerged as a significant focus of participants’ inquiries. There was a strong desire for information on the clinical application of GONS, its side-effects profile, and potential interactions with concurrent therapies like Deep Brain Stimulation (DBS). Participants voiced apprehension about neurological side effects, including increased headaches or tremors, risks of overstimulation, and withdrawal symptoms if the device was discontinued. Some questioned whether the treatment might interfere with sleep or exacerbate existing conditions, as one migraine sufferer remarked, “I don’t want extra headaches!” Other concerns included the ethical standards underpinning the intervention and its safety for human use.

Overall, the findings highlight participants’ interest in GONS as a treatment option for cognitive symptoms in PD, tempered by concerns about safety, efficacy, and practical aspects of its use. The qualitative responses underscore the need for clear, accessible information to address gaps in understanding and enhance patient confidence in adopting this novel intervention.

### Stage 2: Open label field study

***Participant characteristics***

**Figure 1.**
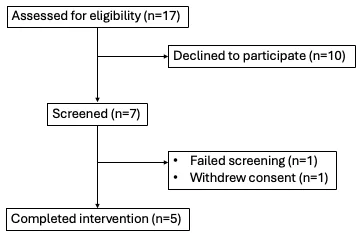
Recruitment flowchart for ONS intervention study.

Five participants with PD-MCI completed the open-label field study ( Figure 1). The cohort included three males and two females, with a mean age of 67.2 years (range 56–82). All participants were retired or unemployed due to health-related reasons. One participant reported that their PD symptoms had a mild impact on daily functioning, three reported a moderate impact, and one reported severe functional impairment.

***Adherence to intervention protocol***

All five participants adhered fully to the intervention protocol, completing three study visits and a follow-up semi-qualitative survey. There were no deviations from the protocol, and all outcome measures were completed by each participant.

***Safety and tolerability***

All participants completed a questionnaire assessing potential adverse effects associated with transcranial direct current stimulation (tDCS) as proposed by Brunoni. Commonly screened adverse effects included headache, neck pain, tingling, scalp pain, itching, burning, skin redness, sleepiness, trouble concentrating, and acute mood changes. No participant reported any adverse events, with all items rated as ‘absent.’

A semi-qualitative post-intervention survey using Likert scales (1–10) evaluated participants’ perceptions of the intervention. Ratings indicated a high degree of safety (mean 10/10) and acceptability (mean 9.4/10). Convenience received a slightly lower average rating (7.4/10), with travel to the study site noted as the primary inconvenience. When asked to rate the likelihood of choosing ONS as a treatment for cognitive issues in PD if found effective, participants provided a unanimous and positive response (mean 9.4/10).

Mood (BDI) and anxiety (BAI) rating scales showed no significant differences pre- and post-intervention ( Table 3).

**Table 3.** Cognitive and neuropsychiatric outcomes measured at visit 1 (day 0) and visit 3 (day 28).

	Visit 1 (mean ± SD)	Visit 3 (mean ± SD)	Mean of differences (95% CI)	SD of differences	*p* value	Cohen’s d
MMSE ^1^	27.2 ± 3.34	28.6 ± 2.6	1.4 (-0.27 to 3.07)	1.3	**0.08**	1.08
BDI ^2^	15.6 ± 10.45	15.2 ± 12.52	-0.4 (-6.8 to 6.0)	5.2	0.87	0.08
BAI ^3^	13 ± 7.75	13 ± 6.29	0.0 (-4.0 to 4.0)	3.2	>0.99	0.0
WAIS DS TRS ^4^	26 ± 3.74	29.8 ± 4.32	3.8 (-1.42 to 9.02)	4.2	0.11	0.9
COWAT ^5^ (letters)	49 ± 14.34	47.4 ± 16.53	-1.600 (-6.377 to 3.177)	3.847	0.41	0.42
COWAT (categories)	16.4 ± 7.23	11.4 ± 3.91	-5.000 (-14.12 to 4.124)	7.348	0.20	-0.68
CLVT-II (TL) ^6^	31 ± 13.27	35.6 ± 12.4	4.6 (1.741 to 7.459)	2.3	**0.01**	2.0
Trail-making	50.35 ± 15.72	46.34 ± 23.16	-4.01 (-25 to 17)	17	0.62	-0.24

^1^Mini-mental state examination; ^2^Beck Depression Inventory; ^3^Beck Anxiety Inventory; ^4^Weschler Adult Intelligence Scale, Digit Span, Total Recognition Score; ^5^Controlled Oral Word Association Test; ^6^California Verbal Learning Test II (Total Learning)

One participant mentioned that the typing component of the word association task was challenging due to Parkinsonian tremor, which impacted performance.

***Preliminary evidence of efficacy***

**Figure 2.**
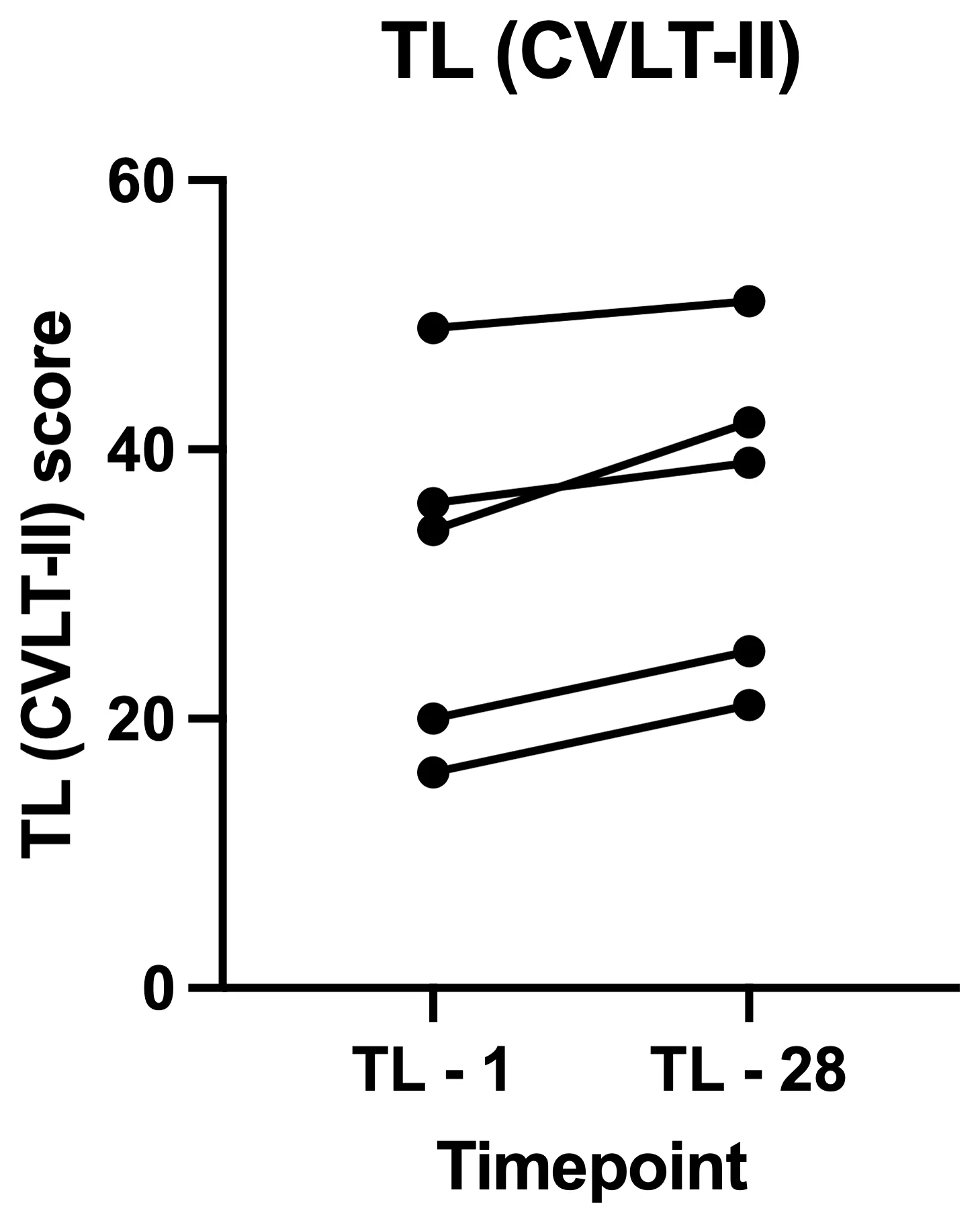
Trend to improvement in Total Learning score on California Verbal Learning Test-II.

**Figure 3.**
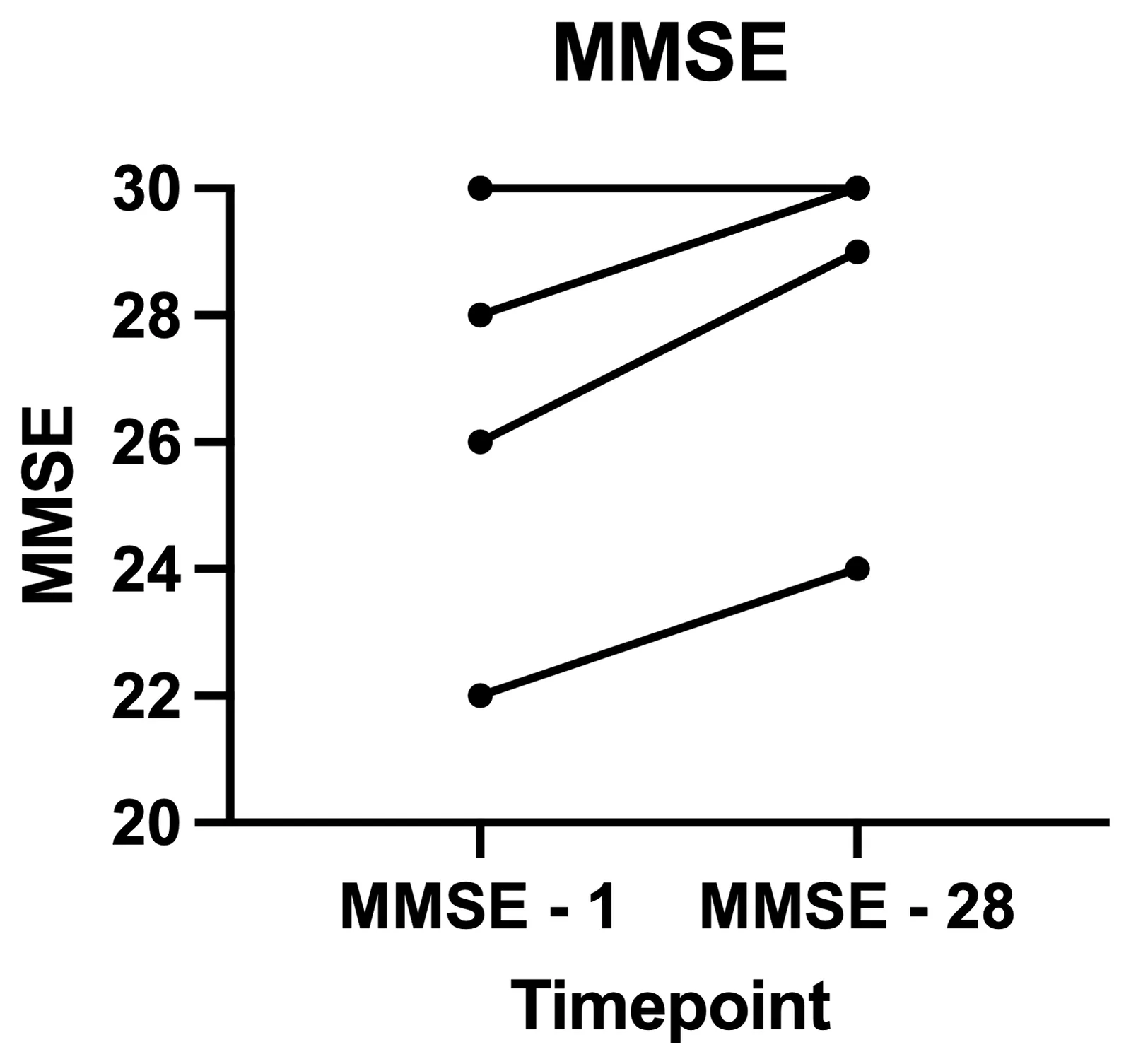
Trend to improvement in Mini Mental State Examination score.

In this small feasibility trial, five participants completed the intervention, and no adverse events were reported, indicating that the intervention was well-tolerated. Descriptive analysis of cognitive outcomes suggested a potential trend toward improvement in total learning (mean difference = 4.6, 95% CI: 1.7 to 7.5, d = 2.0), though this was not statistically significant ( Figure 2). Similarly, trends toward improvements in the Mini-Mental State Examination (MMSE; Figure 3) and the WAIS digit span were observed, but these did not reach statistical significance.

## Discussion

This two-stage study provides preliminary support for the feasibility of GONS as a potential intervention for cognitive impairment in PD-MCI. Stage 1, which involved a survey examining the knowledge, attitudes, and practices of individuals with PD regarding non-pharmacological interventions, revealed that cognitive impairment is often under-recognized and undertreated in clinical practice, particularly when compared to motor symptoms. Many participants reported cognitive changes since their PD diagnosis, and following an informational video, a large majority expressed openness to exploring non-invasive neuromodulation as a treatment for cognitive symptoms. These findings align with existing literature that emphasizes the cognitive aspects of PD as a largely overlooked domain in both clinical care and research ^3^.

The qualitative results from the survey provided deeper insights into participants' views on non-drug treatments like GONS. Several respondents emphasized that cognitive issues in PD are often treated as secondary to motor symptoms, which reflects a gap in both clinical discussions and research addressing cognitive concerns in this patient population. Participants expressed interest in the potential for non-pharmacological treatments to alleviate these symptoms. While some reported positive experiences with similar treatments, such as functional electrical stimulation for gait, others raised concerns based on prior negative encounters with electrostimulation, such as nerve conduction studies or electroconvulsive therapy. This range of feedback underscores the importance of addressing individual patient experiences and perceptions, which can vary significantly when it comes to neuromodulatory interventions.

A key concern expressed by participants was the possibility of overstimulation during GONS sessions, as well as potential withdrawal or tolerance effects. These insights suggest that future studies should incorporate comprehensive educational materials about ONS, including a Frequently Asked Questions (FAQ) section addressing common concerns about the treatment, its side effects, and its interactions with other therapies like deep brain stimulation. Clear, accessible information is essential, particularly as neuromodulation research continues to evolve. Despite concerns, participants largely recognized the potential for ONS to improve daily functioning, with many hopeful that it could help individuals with PD maintain full-time employment and engage more effectively in daily activities. These findings are critical for guiding future research and improving patient-centred care in PD.

Stage 2 of the study involved an open-label field trial to assess the feasibility and acceptability of an GONS protocol adapted from a concurrent trial in individuals with amnestic MCI. Five participants completed the trial, and all expressed strong positive impressions of the intervention. The safety and acceptability ratings were high, and all participants indicated that they would be very likely to consider GONS or a similar non-invasive neuromodulatory intervention if it was proven to be effective. One participant with more severe PD symptoms noted that their tremor interfered with fine motor tasks, such as typing, highlighting the potential impact of motor symptoms on the measurement of cognitive outcomes. This observation suggests that future protocols should account for the confounding effects of motor symptoms, such as tremor and bradykinesia, which could affect cognitive performance assessments in the PD population.

While the overall acceptability of GONS was rated at 9.4 on average by all participants, the convenience of the intervention scored lower at 7.4. The primary driver for this was felt to be the necessity of travelling to the study site to take part. Future studies utilising GONS may consider setting up participants to receive stimulation at home, either with visits from study staff or after receiving training in self-administration.

This study demonstrates that GONS is not only feasible but also well-accepted by individuals with PD-MCI. The open-label trial showed that the GONS intervention protocol is both safe and tolerable for this patient group, providing a foundation for future randomized controlled trials. Given the promising safety and acceptability data, future research could focus on refining the protocol to better mitigate the impact of motor symptoms on measurement of cognitive performance. As highlighted by a recent Cochrane review, there is a clear need for effective non-pharmacological interventions in Parkinson’s disease (PD). Pharmacological treatments offer only modest benefits and are often limited by side effects, polypharmacy, and comorbidities, which compromise their tolerability and accessibility ^2, 23, 24^.

This study is not without limitations. The self-selection bias inherent in both stages of the study may have influenced the findings, as individuals with clear cognitive symptoms or heightened interest in potential treatments may have been more likely to participate. This bias may limit the generalizability of the results. Additionally, the survey sample was relatively homogeneous and non-representative, comprising mostly individuals who are well-educated, technologically proficient, and engaged with patient organizations in Ireland and the UK. To assess potential sampling bias and the presence of predictor variable, we examined the data to assess whether there were any significant predictors of responses. No significant associations were identified, suggesting limited evidence of bias in current sample. However, considering the small sample size, the study may have been underpowered to detect subtle effects. Future studies with a larger and more diverse sample pool will be better suited to conducting stratified analyses and explore subgroup differences and potential biases.

This specific recruitment approach may not reflect the broader PD population, limiting the external validity of the results. Moreover, the survey tool itself was specifically developed for this study, and although it was modelled after existing tools in the literature, none of the instruments used were validated questionnaires. The reliance on self-reported data further introduces potential bias, particularly in the section concerning cognitive issues in PD. The subjective nature of self-reports on cognitive symptoms introduces ambiguity regarding their true presence and severity, and it is unclear whether factors other than PD may have contributed to the reported cognitive difficulties.

For Stage 2, the adaptation of the intervention protocol from a trial in individuals with amnestic MCI may have contributed to some limitations. The Mini-Mental State Examination (MMSE), used as the baseline measure of cognitive function, is primarily designed to screen for amnestic deficits and may not be the optimal tool for assessing cognitive function in PD-MCI, where impairments in executive function and visuospatial processing are more common. Future studies might consider using alternative measures, such as the Montreal Cognitive Assessment (MoCA) or the Addenbrooke’s Cognitive Examination III (ACE-III), which are more sensitive to the types of cognitive deficits seen in PD.

As stage 2 was a small, open-label study focused primarily on safety and tolerability, any evidence of efficacy must be interpreted with caution. Given the lack of control or comparator group, the possibility of a learning or repeated exposure effect exists, although there is evidence to support that the MMSE has good test-retest reliability overall ^25^. The efficacy of GONS for improving cognitive function in PD remains to be explored in larger, randomized controlled trials, stemming from this initial safety and tolerability study. Similarly, future trials might explore a longer timeframe for follow up in terms of tolerability.

## Conclusion

This study underscores that cognitive impairment is an under-recognized aspect of PD, with patients expressing interest in novel neuromodulatory treatments due to limited efficacy of pharmacological options. In an open-label study, all five participants who completed GONS treatment found it safe, comfortable, and without adverse effects, with one cognitive outcome showing improvement and a non-significant increase in MMSE scores. The findings support further exploration of neuromodulatory interventions for PD-related cognitive impairment. Participant feedback highlights the need to adapt protocols for PD patients, considering motor symptoms that may hinder tasks like typing. Future randomized sham-controlled trials are necessary to assess efficacy with adequate statistical power.

## Ethical approval statement

The study was conducted in accordance with the Declaration of Helsinki, and approved by the Health Sciences Ethics Committee of Trinity College Dublin (for stage 1 of the study, approval granted 1 ^st^ of November 2022, reference number 220605) and the Ethics Committee of The School of Psychology, Trinity College Dublin (for stage 2 of the study, approval granted 25 ^th^ of September 2024, approval ID SPREC102021-23).

## Informed consent statement

Written Informed consent was obtained from all subjects involved in the study.

## Abbreviations

The following abbreviations are used in this manuscript:

PD                 Parkinson’s disease

MCI             Mild cognitive impairment

PDD            Parkinson’s disease dementia

tDCS           Transcranial direct-current stimulation

rTMS          Repetitive transcranial magnetic stimulation

DBS             Deep brain stimulation

VNS            Vagus nerve stimulation

GONS         Greater Occipital nerve stimulation

UK               United Kingdom

LBD             Lewy body disease

MMSE        Mini mental state exam

MoCA         Montreal cognitive assessment

ACE-III      Addenbrooke’s cognitive examination III

## Data Availability

1.  Raw data: Figshare: “PD ONS tDCS and survey data” https://doi.org/10.6084/m9.figshare.28695155.v3 ^26^ This project contains the following underlying data: PD dataset (neuropsychological and tolerability data for ONS intervention) Survey data (results of survey on attitudes to ONS treatment) Data is available under the terms of the CC BY 4.0 licence 2.  Extended data: Figshare: “ONS tCDS and survey study extended data” **https://figshare.com/articles/figure/GONS_survey/30009895/2** ^27^ This project contains the following underlying data: Survey questionnaire (survey provided to survey participants) Survey consent form (consent form provided to survey participants) GONS Participant information leaflet (PI leaflet provided to GONS participants) GONS phone screen form (screening form GONS debriefing sheet GONS exit questionnaire/adverse effects Data is available under the terms of the CC BY 4.0 licence
