# Supplementary material for: Greater occipital nerve stimulation for cognitive impairment in Parkinson's disease: A two-stage feasibility and tolerability study
Source: HRB Open Res. 2026 Jun 30;8:124. Originally published 2025 Nov 21. [Version 2] doi: 10.12688/hrbopenres.14230.2 (PMC13554840; doi:10.12688/hrbopenres.14230.2)
Supplement: Peer review history

# Reviewer report: Reviewer response for version 2

**DOI:** 10.21956/hrbopenres.15974.r56301

**Published:** 2026-07-15

**Authors:** Patricia C. Heyn

**Affiliations:** 1 University of Colorado Anschutz Medical Campus, Aurora, CO, USA

The authors satisfactory addressed my concerns.

Is the work clearly and accurately presented and does it cite the current literature?

Partly

If applicable, is the statistical analysis and its interpretation appropriate?

No

Are all the source data underlying the results available to ensure full reproducibility?

Partly

Is the study design appropriate and is the work technically sound?

No

Are the conclusions drawn adequately supported by the results?

Partly

Are sufficient details of methods and analysis provided to allow replication by others?

No

Reviewer Expertise:

Alzheimer's disease, neurodegenerative diseases, rehabilitation, brain health, health promotion.

I confirm that I have read this submission and believe that I have an appropriate level of expertise to confirm that it is of an acceptable scientific standard.

# Reviewer report: Reviewer response for version 1

**DOI:** 10.21956/hrbopenres.15651.r51799

**Published:** 2026-01-08

**Authors:** Patricia C. Heyn

**Affiliations:** 1 University of Colorado Anschutz Medical Campus, Aurora, CO, USA

This manuscript reports a two-stage mixed-methods feasibility study evaluating greater occipital nerve stimulation (GONS) using transcranial direct current stimulation (tDCS) as a potential intervention for cognitive impairment in Parkinson’s disease (PD). 

 The stage 1 provides useful data to justify proceeding to feasibility testing, and the stage 2 is supposed to emphasize safety, tolerability, and acceptability.

 Concerns:

 1) A major issue with this study is the sample selection and very small size.  Sample size justification is absent, particularly for Stage 2 (n=5).

 2) Although participants for phase 2 were selected from a PD clinic, there is no information ensuring that they were clinically diagnosed with PD or if it was self-reporting information.  Diagnostic criteria for “Lewy body-related MCI” are not explicitly stated (e.g., Movement Disorder Society PD-MCI criteria).

 3) The reporting is too ambitious for this rudimentary feasibility study (e.g., “trend toward improvement,” large effect sizes).

 4) Information exploring the practice effects on repeated cognitive testing (e.g., CVLT-II, MMSE) is not sufficiently incorporated in the discussion section.

 5) There are no stopping rules or predefined feasibility thresholds described.

 6) For the qualitative analysis, the number of participants contributing to each theme is not reported.

 Please address the following concerns:

1. Describe participants selection procedures for Stage 2.
2. Add justification for sample size (e.g., pragmatic constraints, precedent in pilot neuromodulation studies).
3. How participants were diagnosed and the diagnostic criteria used for PD-MCI / Lewy body-related MCI.
4. Describe how many participants contributed to each qualitative theme.
5. Conclusions regarding safety are preliminary and limited by sample size, explain how these limitations impact the study results.
6. Provide sufficient methodological and procedural detail to ensure reproducibility such  as participant selection rationale for Stage 2, training and standardization of outcome assessments, and how authors handled missing data.
7. Expand discussion of practice effects, repeated testing, and absence of a control group.
8. Revise conclusions to focus strictly on feasibility, acceptability, and very preliminary safety data.
9. Remove or substantially temper the language suggesting efficacy.
10. Emphasize rigor for future research such as that randomized, sham-controlled trials are required before any conclusions about benefit to the population can be drawn.
11. Make sure to acknowledge all the limitations (e.g. design, very small sample size, absence of control group, and the strong likelihood of practice effects)

Is the work clearly and accurately presented and does it cite the current literature?

Partly

If applicable, is the statistical analysis and its interpretation appropriate?

No

Are all the source data underlying the results available to ensure full reproducibility?

Partly

Is the study design appropriate and is the work technically sound?

No

Are the conclusions drawn adequately supported by the results?

Partly

Are sufficient details of methods and analysis provided to allow replication by others?

No

Reviewer Expertise:

Alzheimer's disease, neurodegenerative diseases, rehabilitation, brain health, health promotion.

I confirm that I have read this submission and believe that I have an appropriate level of expertise to confirm that it is of an acceptable scientific standard, however I have significant reservations, as outlined above.

# Response

**Published:** 2026-06-19

**Authors:** Liam Kennedy

**Affiliations:** Psychiatry, Trinity College Dublin School of Medicine, Dublin, Leinster, Ireland

HRB Open Research Article 14230 - Greater occipital nerve stimulation for cognitive impairment in Parkinson's disease: A two-stage feasibility and tolerability study

**Reviewer 3**

*Author comments*

 The authors would like to thank the reviewer for their careful analysis and constructive feedback regarding our manuscript. Below we have outlined our responses to the concerns raised by the reviewer.

**1. Describe participants selection procedures for Stage 2.**

 We thank the reviewer for highlighting this omission in our manuscript, and have added specific diagnostic detail added to participant selection procedures, including use of MDS PD-MCI criteria

**2. Add justification for sample size (e.g., pragmatic constraints, precedent in pilot neuromodulation studies).**

 We have updated the manuscript to outline the pragmatic constraints leading to a total sample number of 5 out of 17 potentially recruited participants.

**3. How participants were diagnosed and the diagnostic criteria used for PD-MCI / Lewy body-related MCI.**

 As in point 1 above, all participants were patients with PD as diagnosed by a movement disorder specialist, and who were attending a subspecialist clinic for Lewy body-related neuropsychiatric or cognitive issues. All participants met criteria for PD-MCI as per MDS.

**4. Describe how many participants contributed to each qualitative theme.**

 We agree that this is an important omission and have updated the manuscript to include the specific number of participants contributing to each theme.

**5. Conclusions regarding safety are preliminary and limited by sample size, explain how these limitations impact the study results.**

 With thanks, we have updated the manuscript to place much more emphasis on the preliminary and exploratory nature of these results

**6. Provide sufficient methodological and procedural detail to ensure reproducibility such as participant selection rationale for Stage 2, training and standardization of outcome assessments, and how authors handled missing data.**

 The methods section of Stage 2 has been substantially updated with more detail on the areas highlighted. No imputation procedures were required as no missing data were identified.

**7. Expand discussion of practice effects, repeated testing, and absence of a control group.**

 Our updated manuscript includes more robust discussion of these limitations and adds specific references to studies demonstrating the practice effect evident in the MMSE and CVLT-II, as well as methodological suggestions for future studies to avoid or minimise a practice effect.

**8. Revise conclusions to focus strictly on feasibility, acceptability, and very preliminary safety data.**

 We have removed any discussion of cognitive outcomes from the conclusions section.

**9. Remove or substantially temper the language suggesting efficacy.**

 The manuscript has been updated throughout to this effect, with thanks

**10. Emphasize rigor for future research such as that randomized, sham-controlled trials are required before any conclusions about benefit to the population can be drawn.**

 We have added such further recommendations for future research to the discussion section

**11. Make sure to acknowledge all the limitations (e.g. design, very small sample size, absence of control group, and the strong likelihood of practice effects)**

 The above limitations have been more strongly emphasised throughout the manuscript with thanks

# Reviewer report: Reviewer response for version 1

**DOI:** 10.21956/hrbopenres.15651.r51972

**Published:** 2026-01-05

**Authors:** Jean-Paul Nguyen

**Affiliations:** 1 Clinique Bretéché, Nantes, France

The article entitled ‘Greater occipital nerve stimulation for cognitive impairment in Parkinson's disease: A two-stage feasibility and tolerability study’ addresses the issue of the role of locus coeruleus (LC) dysfunction in the development of cognitive disorders. In this article, the author focuses on the cognitive disorders encountered in Parkinson's disease. He hypothesizes that stimulation of the occipital nerves stimulates LC function and could therefore improve cognitive disorders. The cause-and-effect relationship could be established by studying ‘neuromelanin MRI’ and analyzing salivary α-amylase levels.

 The first comment concerns the usefulness of the preliminary study on the acceptability of the procedure, given that it is a non-invasive procedure and its safety has been reported in numerous publications. I think this chapter is of little interest and even adds to the confusion, as Parkinson's patients and patients with Lewy body disease are mixed. Qualitative analysis is interesting in principle, but for me it is particularly interesting after treatment to find out about patients' experiences of the procedure and its results. It is a pity that the analysis of the results of five treated patients is not a true qualitative analysis with interviews, but rather responses to questionnaires.

 In the introduction, the author rightly reports that invasive and non-invasive neuromodulation techniques have proven to be of little or no effect on cognitive disorders, but he could have cited a 2019 publication reporting a significant improvement in cognitive disorders through the use of tACS (Del Felice A, Castiglia L, Formaggio E, Cattelan M, Scarpa B, Manganotti P, Tenconi E, Masiero S. Personalised transcranial alternating current stimulation (tACS) and physical therapy to treat motor and cognitive symptoms in Parkinson's disease: A randomised cross-over trial. Neuroimage Clin. 2019;22:101768. doi: 10.1016/j.nicl.2019.101768. Epub 2019 Mar 18. PMID: 30921609; PMCID: PMC6439208). In this study, tACS sessions lasted 30 minutes and were repeated 10 times over 15 consecutive days. This contrasts with the protocol used in this study, which consisted of a single 4-minute stimulation, if I understand correctly. It is almost obvious that we cannot expect to obtain any positive results with such a brief protocol. Furthermore, the protocol itself is not described. Is there an anode on each side (bianodal stimulation) or an anode on one side (P3) and a cathode on the other (P4)? A diagram would be welcome.

 There is no mention of an ethics committee.

 In my opinion, the article should be summarized in a letter to the editor reporting the five cases treated and opening up a future protocol including neuromelanin MRI and salivary α-amylase testing.

Is the work clearly and accurately presented and does it cite the current literature?

Partly

If applicable, is the statistical analysis and its interpretation appropriate?

Not applicable

Are all the source data underlying the results available to ensure full reproducibility?

Yes

Is the study design appropriate and is the work technically sound?

Partly

Are the conclusions drawn adequately supported by the results?

Partly

Are sufficient details of methods and analysis provided to allow replication by others?

No

Reviewer Expertise:

Pain, Neurosurgery, Non Invasive Brain Stimulation.

I confirm that I have read this submission and believe that I have an appropriate level of expertise to confirm that it is of an acceptable scientific standard, however I have significant reservations, as outlined above.

# Response

**Published:** 2026-06-19

**Authors:** Liam Kennedy

**Affiliations:** Psychiatry, Trinity College Dublin School of Medicine, Dublin, Leinster, Ireland

HRB Open Research Article 14230 - Greater occipital nerve stimulation for cognitive impairment in Parkinson's disease: A two-stage feasibility and tolerability study

**Reviewer 2**

*Author comments*

 The authors would like to thank the reviewer for their careful analysis and constructive feedback regarding our manuscript. Below we have outlined our responses to the points raised by the reviewer.

**1. The first comment concerns the usefulness of the preliminary study on the acceptability of the procedure, given that it is a non-invasive procedure and its safety has been reported in numerous publications**. **“**I think this chapter is of little interest and even adds to the confusion, as Parkinson's patients and patients with Lewy body disease are mixed. Qualitative analysis is interesting in principle, but for me it is particularly interesting after treatment to find out about patients' experiences of the procedure and its results. It is a pity that the analysis of the results of five treated patients is not a true qualitative analysis with interviews, but rather responses to questionnaires”

 We thank the reviewer for their thoughts. The suggestion for fully qualitative interviews post-procedure is accepted with thanks and has been added to the discussion section in relation to possible future studies.

 2. **In the introduction, the author rightly reports that invasive and non-invasive neuromodulation techniques have proven to be of little or no effect on cognitive disorders, but he could have cited a 2019 publication reporting a significant improvement in cognitive disorders through the use of tACS** (Del Felice A, Castiglia L, Formaggio E, Cattelan M, Scarpa B, Manganotti P, Tenconi E, Masiero S. Personalised transcranial alternating current stimulation (tACS) and physical therapy to treat motor and cognitive symptoms in Parkinson's disease: A randomised cross-over trial. Neuroimage Clin. 2019;22:101768. doi: 10.1016/j.nicl.2019.101768. Epub 2019 Mar 18. PMID: 30921609; PMCID: PMC6439208). In this study, tACS sessions lasted 30 minutes and were repeated 10 times over 15 consecutive days. This contrasts with the protocol used in this study, which consisted of a single 4-minute stimulation, if I understand correctly. It is almost obvious that we cannot expect to obtain any positive results with such a brief protocol. Furthermore, the protocol itself is not described. Is there an anode on each side (bianodal stimulation) or an anode on one side (P3) and a cathode on the other (P4)? A diagram would be welcome”

 The Del Felice study mentioned adds valuable background and has been added to the introduction as additional context for our study with thanks. The following specific detail on the stimulation protocol has been added to the methods, with reference to the original intervention protocol referred to in the manuscript (Adcock KS, Lawlor B, Robertson IH, et al.: Diminishing accelerated long-term forgetting in Mild Cognitive Impairment: study protocol for a prospective, double-blind, placebo-controlled, randomized controlled trial. *Contemp Clin Trials Commun.* 2022;30: 100989. 36117569 10.1016/j.conctc.2022.100989 9478352)

 “All stimulation sessions were delivered by the same investigator, who received training in the use of transcranial direct current stimulation devices and followed a standardized operating procedure. GONS using direct current was transmitted via a saline-soaked pair of surface sponges (35 cm ^2^) and delivered by specially developed, battery-driven, constant current stimulator with a maximum output of 10 mA (Eldith; www.neuroconn.de). For each participant receiving GONS, the anodal electrode was placed over the left C2 nerve dermatome, and cathodal electrode placed over the right C2 dermatome. A constant current of 1.5 mA was applied for 250 seconds, with a ramp up period of 30 seconds and ramp down period of 5 seconds”

**3. There is no mention of an ethics committee.**

 The original manuscript includes a statement on ethical approval towards the end of the manuscript, following the discussion.

# Reviewer report: Reviewer response for version 1

**DOI:** 10.21956/hrbopenres.15651.r51806

**Published:** 2025-12-27

**Authors:** Moaz Elsayed Abouelmagd

**Affiliations:** 1 Cairo University, Cairo, Egypt

This two stage study evaluates Greater Occipital Nerve Stimulation (GONS) using tDCS as a potential intervention for cognitive impairment in Parkinson disease. The topic is clinically interesting and fits well within early feasibility work, but there are several methodological and interpretive issues that should be addressed.

 1. Sample size, stopping rules, and conclusions about safety

 Stage 2 includes only 5 participants, and there is no explanation of how this number was chosen or whether any stopping rule was planned. Even for a feasibility study, a brief justification is needed, for example linking the sample size to pragmatic constraints or to previous pilot protocols. In addition, such a small sample cannot meaningfully support firm conclusions about safety. The text should clarify that safety conclusions are preliminary and based on limited numbers.

 2. Diagnostic criteria for Lewy body related MCI

 The manuscript states that participants had Lewy body related MCI, but the diagnostic criteria are not clearly described. 

 3. 

 In a very small feasibility sample, the use of a wide battery of cognitive outcomes greatly increases the risk of type I error. The results should be presented descriptively only (I will clarify that downward) but the p values in Table 3 may be overinterpreted by readers. Explicitly state that all analyses are exploratory and hypothesis generating.

 Consider removing p values altogether and presenting the study as descriptive.

 4. Practice effects on CVLT II and MMSE in the absence of a control arm

 The CVLT II is a list learning task in which the patient learns a list of 16 words. When patients take the same test more than once, they often recall both the specific words and the strategy from the first session, even weeks later. Without a control arm, any apparent improvement cannot be separated from practice effects, which may account for a large part of the change.

 Research has shown that healthy adults and even patients with mild cognitive impairment can recall on average about 8 more words in total simply by repeating the test, which could easily inflate p values and give the impression of meaningful change in such a small sample:

 Woods SP, Delis DC, Scott JC, Kramer JH, Holdnack JA. The California Verbal Learning Test second edition: test retest reliability, practice effects, and reliable change indices for the standard and alternate forms. Arch Clin Neuropsychol. 2006;21(5):413 420. doi:10.1016/j.acn.2006.06.002

 A similar practice effect has been described for the MMSE:

 Galasko D, Abramson I, Corey Bloom J, Thal LJ. Repeated exposure to the Mini Mental State Examination and the Information Memory Concentration Test results in a practice effect in Alzheimer's disease. Neurology. 1993;43(8):1559 1563. doi:10.1212/wnl.43.8.1559

 These points should be discussed more explicitly, and the potential impact of practice effects on the observed changes should be acknowledged as a key limitation and no conclusions shall be made on efficacy.

 5. 

 The manuscript currently focuses on change between phases 1 and 3. Given the strong practice effect concerns, it would be more informative to present data from all three test phases so that readers can see the full trajectory. Ideally, patients would have been tested two or three times before the intervention to allow the main practice effect to occur before baseline; this could be mentioned in the discussion as a methodological improvement for future work.

 6. 

 For small single arm feasibility studies, group averages are difficult to interpret. I strongly recommend  present the cognitive outcomes at the individual patient level rather than only as group means. This would allow readers to judge whether any patients showed credible individual improvement.

 7. 

 The manuscript describes a "trend toward improvement" in CVLT II total learning with a large Cohen d. Given the very small sample size, open label design, and strong practice effect concerns, this language should be made more cautious and clearly framed as descriptive. I suggest avoiding wording that implies efficacy and instead stressing that results are preliminary signals that require confirmation in a larger, controlled trial.

 8. Limitations and generalisability

 The limitations section appropriately notes self-selection and a relatively homogeneous sample. This could be expanded by pointing out that recruitment through support groups and a specialist clinic may bias toward patients who are more engaged, more health literate, and perhaps more willing to consider novel interventions such as GONS. 

 9. 

 For the qualitative component, it would be helpful to indicate how many participants contributed to each theme, even approximately.

Is the work clearly and accurately presented and does it cite the current literature?

Partly

If applicable, is the statistical analysis and its interpretation appropriate?

No

Are all the source data underlying the results available to ensure full reproducibility?

Partly

Is the study design appropriate and is the work technically sound?

Partly

Are the conclusions drawn adequately supported by the results?

No

Are sufficient details of methods and analysis provided to allow replication by others?

Partly

Reviewer Expertise:

Neurology, biostatstics

I confirm that I have read this submission and believe that I have an appropriate level of expertise to confirm that it is of an acceptable scientific standard, however I have significant reservations, as outlined above.

# Response

**Published:** 2026-06-19

**Authors:** Liam Kennedy

**Affiliations:** Psychiatry, Trinity College Dublin School of Medicine, Dublin, Leinster, Ireland

HRB Open Research Article 14230 - Greater occipital nerve stimulation for cognitive impairment in Parkinson's disease: A two-stage feasibility and tolerability study

**Reviewer 1**

*Author comments*

 The authors would like to thank the reviewer for their careful analysis and constructive feedback regarding our manuscript. Below we have outlined our responses to some of the points raised by the reviewer.

**1. Sample size, stopping rules, and conclusions about safety**

 “Stage 2 includes only 5 participants, and there is no explanation of how this number was chosen or whether any stopping rule was planned. Even for a feasibility study, a brief justification is needed, for example linking the sample size to pragmatic constraints or to previous pilot protocols. In addition, such a small sample cannot meaningfully support firm conclusions about safety. The text should clarify that safety conclusions are preliminary and based on limited numbers”

 We agree that further justification of the small sample size for this feasibility study would be helpful and have added more detail on the pragmatic recruitment constraints that lead to this sample size. We have increased emphasis on the preliminary nature of our results.

**2. Diagnostic criteria for Lewy body related MCI**

 “The manuscript states that participants had Lewy body related MCI, but the diagnostic criteria are not clearly described”

 We agree that this is an important detail and have included the specific criteria used (MDS PD-MCI criteria) with thanks.

**3. In a very small feasibility sample, the use of a wide battery of cognitive outcomes greatly increases the risk of type I error.**“The results should be presented descriptively only (I will clarify that downward) but the p values in Table 3 may be overinterpreted by readers. Explicitly state that all analyses are exploratory and hypothesis generating. Consider removing p values altogether and presenting the study as descriptive”

 We have added further emphasis on any results related to cognitive outcomes being descriptive only and non-significant statistically. We thank the reviewer for their suggestion and have removed p values and effect sizes from table 3.

**4. Practice effects on CVLT II and MMSE in the absence of a control arm**

 “The CVLT II is a list learning task in which the patient learns a list of 16 words. When patients take the same test more than once, they often recall both the specific words and the strategy from the first session, even weeks later. Without a control arm, any apparent improvement cannot be separated from practice effects, which may account for a large part of the change.

 Research has shown that healthy adults and even patients with mild cognitive impairment can recall on average about 8 more words in total simply by repeating the test, which could easily inflate p values and give the impression of meaningful change in such a small sample. A similar practice effect has been described for the MMSE. These points should be discussed more explicitly, and the potential impact of practice effects on the observed changes should be acknowledged as a key limitation and no conclusions shall be made on efficacy.Ideally, patients would have been tested two or three times before the intervention to allow the main practice effect to occur before baseline; this could be mentioned in the discussion as a methodological improvement for future work”

 We thank the reviewer for this helpful consideration. While we had addressed the potential practice effect of the MMSE in our original manuscript, we have added more explicit discussion of this in terms of both cognitive measures and highlighted this as a limitation to our study design, to be considered for future studies.

**5. The manuscript currently focuses on change between phases 1 and 3.** “Given the strong practice effect concerns, it would be more informative to present data from all three test phases so that readers can see the full trajectory”

 By design, data was only collected at visit 1 and visit 3, as described in the methodology. However, we agree that more granular data capturing any progressive change would be interesting and we would consider this in any future studies.

**6. For small single arm feasibility studies, group averages are difficult to interpret.** “I strongly recommend  present the cognitive outcomes at the individual patient level rather than only as group means. This would allow readers to judge whether any patients showed credible individual improvement”

 Following discussion, we agree that individual-level data is worth presenting and we have added table 4 to this effect.

**7. The manuscript describes a "trend toward improvement" in CVLT II total learning with a large Cohen d.** “Given the very small sample size, open label design, and strong practice effect concerns, this language should be made more cautious and clearly framed as descriptive. I suggest avoiding wording that implies efficacy and instead stressing that results are preliminary signals that require confirmation in a larger, controlled trial”

 We have significantly updated the language describing our results to emphasise its statistical non-significance and lack of credibility as evidence for any efficacy signal. We have clearly framed all cognitive outcomes as descriptive.

**8. Limitations and generalisability**“The limitations section appropriately notes self-selection and a relatively homogeneous sample. This could be expanded by pointing out that recruitment through support groups and a specialist clinic may bias toward patients who are more engaged, more health literate, and perhaps more willing to consider novel interventions such as GONS”

 We have updated the manuscript to expand upon the limitations in relation to selection bias as suggested, with thanks.

**9. For the qualitative component, it would be helpful to indicate how many participants contributed to each theme, even approximately.**

 We have updated the qualitative results with clear accounts of the numbers of patients contributing to each listed theme, with thanks.
